# Caveolin-1 Phosphorylation Is Essential for Axonal Growth of Human Neurons Derived From iPSCs

**DOI:** 10.3389/fncel.2019.00324

**Published:** 2019-07-17

**Authors:** Shanshan Wang, Zheng Zhang, Angels Almenar-Queralt, Joseph Leem, Celine DerMardirossian, David M. Roth, Piyush M. Patel, Hemal H. Patel, Brian P. Head

**Affiliations:** ^1^Veterans Affairs San Diego Healthcare System, San Diego, CA, United States; ^2^Department of Anesthesiology, UC San Diego, La Jolla, CA, United States; ^3^Department of Cellular and Molecular Medicine, Sanford Consortium for Regenerative Medicine, La Jolla, CA, United States; ^4^Department of Immunology and Microbial Sciences, The Scripps Research Institute, La Jolla, CA, United States; ^5^Department of Cell and Molecular Biology, The Scripps Research Institute, La Jolla, CA, United States

**Keywords:** NPCs, iPSCs, caveolin-1, phosphorylation, Rac1/Cdc42, axonal growth

## Abstract

Proper axonal growth and guidance is essential for neuron differentiation and development. Abnormal neuronal development due to genetic or epigenetic influences can contribute to neurological and mental disorders such as Down syndrome, Rett syndrome, and autism. Identification of the molecular targets that promote proper neuronal growth and differentiation may restore structural and functional neuroplasticity, thus improving functional performance in neurodevelopmental disorders. Using differentiated human neuronal progenitor cells (NPCs) derived from induced pluripotent stem cells (iPSCs), the present study demonstrates that during early stage differentiation of human NPCs, neuron-targeted overexpression constitutively active Rac1 (Rac1CA) and constitutively active Cdc42 (Cdc42CA) enhance expression of P-Cav-1, T-Cav-1, and P-cofilin and increases axonal growth. Similarly, neuron-targeted over-expression of Cav-1 (termed *SynCav1*) increases axonal development by increasing both axon length and volume. Moreover, inhibition of Cav-1(Y14A) phosphorylation blunts Rac1/Cdc42-mediated both axonal growth and differentiation of human NPCs and *SynCav1(Y14A)*-treated NPCs exhibited blunted axonal growth. These results suggest that: (1) *SynCav1-*mediated dendritic and axonal growth in human NPCs is dependent upon P-Cav-1, (2) P-Cav-1 is necessary for proper axonal growth during early stages of neuronal differentiation, and (3) Rac1/Cdc42CA-mediated neuronal growth is in part dependent upon P-Cav-1. In conclusion, Cav-1 phosphorylation is essential for human neuronal axonal growth during early stages of neuronal differentiation.

## Introduction

Proper axonal growth and guidance are crucial for development of functional neuronal networks. Neuronal growth cones, located at the tip of emerging axons, transduce extracellular growth, and inhibitory cues to the underyling cytoskeleton within the axon (Head et al., 2014). Growth cones are enriched in MLRs, discrete plasmalemmal microdomains enriched in cholesterol, glycosphingolipids, and the scaffolding protein caveolin (Cav) (Sekino et al., 2007; Grider et al., 2009; Whitehead et al., 2012). Cav-1 is a cholesterol binding and scaffolding protein within MLRs that organizes signaling complexes such as Src family kinases (SFKs), cytoskeletal tethering proteins, and Rho GTPases (RhoA, Cdc42, and Rac1) that regulate cytoskeletal dynamics (Head et al., 2008, 2014; Pantera et al., 2009; Berta et al., 2011; Stuermer, 2011). Loss or disruption of MLRs from the leading edge results in growth cone collapse and inhibition of neuritogenesis (Whitehead et al., 2012). Previous research from of our group showed that Cav-1 organized growth-promoting signaling complexes within MLRs and regulated neurotrophic receptor signaling pathways (Head et al., 2008, 2011; Egawa et al., 2017; Mandyam et al., 2017). Specifically, neuron-targeted overexpression of Cav-1 (*SynCav1*) enhanced MLR formation, receptor-mediated cAMP production, TrkB receptor expression and signaling, and augmented dendritic arborization in primary neurons *in vitro* (Head et al., 2011). In addition, *in vivo* delivery of *SynCav1* to the hippocampus of adult and aged mice enhanced both MLR-localized TrkB and structural neuroplasticity, improving hippocampal-dependent memory (Egawa et al., 2017; Mandyam et al., 2017). Past findings demonstrated that Cav-1 was widely involved in opioid-induced dendritic growth (Cui et al., 2017), further demonstrating a regulatory role of Cav-1 on various forms of neuroplasticity.

Modulation of cytoskeletal dynamics is important for proper neuronal differentiation. Rho GTPases (RhoA, Cdc42, and Rac1) are key regulators of cytoskeletal dynamics (Hall and Lalli, 2010; Dent et al., 2011). Dysregulation of cytoskeletal dynamics can lead to growth cone collapse and aberrant synaptic connections that occur in many neurodegenerative conditions such as Alzheimer’s disease (AD) (Heredia et al., 2006; Penzes and Vanleeuwen, 2011) and Down syndrome (Jabbour et al., 1992). While RhoA regulates actin stress fiber assembly and focal adhesion sites; Rac1 controls actin filament accumulations and plasmalemmal protrusion in the form of lamellipodia; Cdc42 produces filopodia and neurite outgrowth. Previous work from others showed that Cav-1 modulates Rho GTPase signaling in non-neuronal cells (Baltierrez-Hoyos et al., 2012; Lim et al., 2014). Because Cav-1 has been shown to colocalize and modulate Rho GTPase activity in non-neuronal cells (Cheng et al., 2010; Bassi et al., 2011; Diaz et al., 2014; Williamson et al., 2015; Coelho-Santos et al., 2016; Xu et al., 2016), the present study tested whether Cav-1 was also involved in Rho GTPase-mediated axonal growth and neuronal differentiation in human neurons derived from induced pluripotent stem cells (iPSCs). A better mechanistic understanding of this interplay within neurons could yield potential targets for promoting functional neuroplasticity.

Kawauchi and colleagues recently demonstrated that Cav-1 promoted early neurite maturation in an endocytic-dependent manner (Shikanai et al., 2018). However, whether Cav-1 phosphorylation was necessary for neurite maturation, specifically axonal maturation and guidance, has never been investigated. Using human neuron progenitor cells (NPCs) derived from iPSCs, we first tested the effects of neuron-targeted Cav-1 expression and Rac1/Cdc42 activation on neuronal growth during differentiation. Furthermore, by using a Cav-1 mutant construct *SynCav1 (Y14A)*, that prevents Cav-1 phosphorylation at tyrosine (Y) 14, we demonstrated that Cav-1 phosphorylation was required for both *SynCav1*-mediated and Rac1/Cdc42-mediated neuronal axonal growth and differentiation.

## Materials and Methods

### NPCs Culture and Differentiation

The use of human induced pluripotent stem cells (hiPSC) was approved by the UCSD IRB/ESCRO (project #:1616206ZX). Human differentiated neuronal progenitor cells (NPCs) were previously derived from hiPSC CVB line (RRID: CVCL_1N86, GM25430) (Young et al., 2015). NPCs were cultured on 20 mg/ml poly-L-ornithine (PLO) and 5 mg/ml laminin (both from Sigma) coated plates as previously described (Kassan et al., 2017). NPC medium [DMEM/F12/Glutamax media with N-2 supplement, B-27 supplement, Pen/Strep and basic Fibroblast Growth Factor (bFGF)] were used for culturing NPCs. The incubator was set at 37°C in 5% CO_2_. Medium was changed every 2–3 days and cells were split with Accutase and Accumax (Innovative Cell Technologies) when arrived 100% confluency. bFGF was removed from the medium to induce differentiation. Medium was changed every 2–3 days.

### Chemicals and Antibodies

Ras-related C3 botulinum toxin substrate 1/Cell division control protein 42 homolog activator (#CN02-A, Cytoskeleton, Inc., San Diego, CA, United States), Antibodies used for immunoblotting and immunofluorescence were as follows: P-Cav-1 (#3251), T-Cav-1 (#3267), P-Src (#2101), T-Src (#2108), and GAPDH (#5274) were all from Cell Signaling Technology (San Diego, CA, United States). P-cofilin (sc-271921) was from Santa Cruz Biotech (Santa Cruz, CA, United States), microtubule-associated protein 2 (MAP2) (ab5392) was from Abcam (Cambridge, MA, United States), anti-neurofilament SMI 31 (#801601) was from BioLegend (San Diego, CA, United States). Primary antibodies were visualized using secondary antibodies conjugated to horseradish peroxidase (HRP) (Santa Cruz Biotech, Santa Cruz, CA, United States) and lumigen ECL ultra (MA-100, Lumigen Inc., Southfield, MI, United States). All displayed bands were compared to molecular weight standards (sc-2035, Santa Cruz Biotech, Santa Cruz, CA, United States). The amount of protein per sample was determined using a dye-binding protein assay (Bio-Rad, Hercules, PA, United States). For immunofluorescence, FITC-488, Texas Red-595 and Cy5-647 secondary antibodies were obtained from Molecular Probes (Carlsbad, CA, United States).

### Generation of Neuron-Targeted Genetic Constructs and Cell Transfection

Lentivirus containing synapsin-caveolin-1 (*SynCav1*), Rac1Q61L (*SynRac1CA*, glutamine (Q) 61 to leucine point mutation, constitutively active Rac1), Cdc42Q61L (*SynCdc42CA*, glutamine (Q) 61 to leucine point mutation, constitutively active Cdc42), and caveolin-1 phospho-mutant [*SynCav1(Y14A)*, tyrosine (Y) 14 to alanine (A) point mutation] were generated as previously described (Head et al., 2011). Differentiated NPCs were treated with either control vector (synapsin-red fluorescent protein, *SynRFP* or synapsin-green fluorescent protein, *SynGFP* as indicated) or empty vector on day 3 in culture.

### Drug Application

After seven days of differentiation, neurons were treated with a small molecule [(#CN02-A) dissolved in NPC medium)] that directly activates Rac1/Cdc42 with various doses and time points (1, 2, and 3 units/ml; 5, 15, and 30 min). Untreated cells served as negative control. To measure related protein expression level change, cells were harvested directly after activator treatment and prepared for Western blot.

### Western Blot

Cells were homogenized with cold RIPA lysis buffer followed by 3 cycles of 20-s bursts of sonication at 4°C. The amount of protein per sample was determined using a dye-binding protein assay (Bio-Rad). Electrophoresis was performed on the samples using 4–12% or 10% acrylamide gels (Invitrogen) and transferred to polyvinylidene difluoride membranes (Millipore) by electroelution. Membranes were blocked in blocking solution [20 mM TBS Tween (1%) (TBST) containing 3% bovine serum albumin (BSA)] and then incubated with primary antibodies overnight at 4°C. After 3 washes with TBST, the membrane was incubated with secondary antibody for 1 h at room temperature (RT). Horseradish peroxidase (HRP)-labeled goat anti-rabbit IgG (Santa Cruz Biotech) was used as secondary antibody. After another 3 times wash with TBST to remove the rest secondary antibody, membrane was incubated with ECL reagent (Amersham Pharmacia Biotech, Piscataway, NJ, United States) and prepared for imaging. Bands were compared to molecular weight standards (Santa Cruz Biotech). GAPDH served as the internal reference by which to standardize the other protein bands. The calculated ratio of the control group was normalized to 100%, and the comparisons of other groups to the control group were represented as percentages.

### Immunofluorescence Confocal Microscopy

Neurons were fixed with 2% paraformaldehyde for 10 min at room temperature and then permeabilized in 0.1% Triton X-100 and blocked with 1% BSA/PBS/Tween (0.05%) for 60 min. Next, cells were incubated with primary antibodies in 1% BSA/PBS/Tween (0.05%) for 24–48 h at 4°C followed by incubation with FITC or Alexa conjugated secondary antibody (1:600 to 1:800) for 1 h at room temperature (RT). Stained cells were mounted in mounting medium containing DAPI and imaged with Olympus confocal microscope system (Applied Precision, Inc., Issaquah, WA, United States) that included a Photometrics CCD mounted on a Nikon TE-200 inverted epi-fluorescence microscope. Exposure times were set to the same value for all groups every time. Quantitation of intensity of GTP-Rac1/GTP-Cdc42 was conducted by using Image J (NIH). Quantitation of axonal length and volume was conducted by using Autoneuron, which measures 3D image volume stacks (MBF Bioscience) generated by Neurolucida as previously described (Head et al., 2011); representative images and tracing image are shown in Supplementary Figure S1.

### Statistical Analysis

Data were expressed as means ± SE. All data were analyzed by unpaired *t*-tests or One-Way ANOVA; *post hoc* comparisons were performed by Student Neuman Keuls tests. Significance was set at *p* < 0.05. Statistical analysis and graphs were made using Prism 7 (GraphPad Software, Inc., La Jolla, CA, United States).

## Results

### *SynCav1* Increases Axonal Growth in Early Stage Differentiation of Human NPCs

**FIGURE 1:**
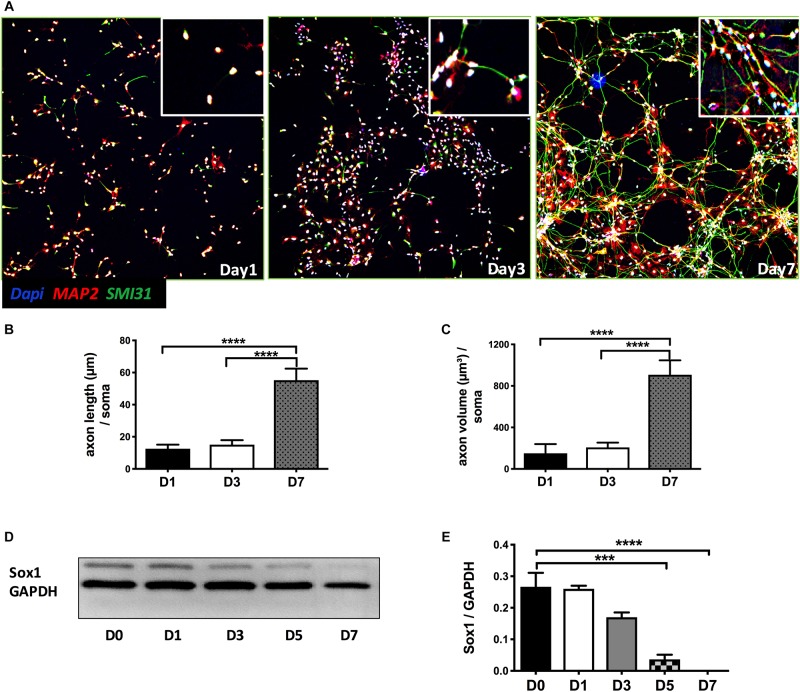
Early stage of differentiation process of NPCs. **(A)** Seven days after bFGF removal, NPCs were fixed and stained for the dendritic and axonal markers MAP2 (green) and SMI 31 (red), respectively. Quantitation of axonal length **(B)** and volume **(C)**. **(D)** NPC lysates at days 0 to 7 differentiation were probed for the stem cell marker SOX1. Quantitation of SOX1 protein expression **(E)**. Data are expressed as mean ± S.E (*n* = 3, ^∗∗∗^*p* < 0.001, ^∗∗∗∗^*p* < 0.0001, One-way ANOVA).

**FIGURE 2:**
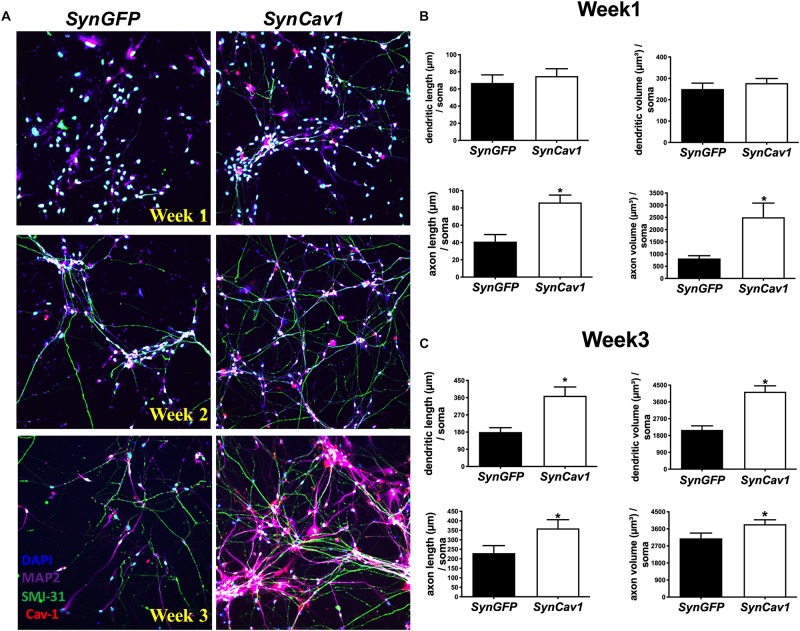
*SynCav1* enhances axonal growth in differentiated NPCs derived from human iPSCs. Differentiated NSCs were transfected with lentiviral *SynCav1* (2 × 10^9^ IU/μl) (or *SynGFP*) on day 3 for 4 days, IF microscopy was performed at 1–3 weeks post transfection. Representative IF images are shown in **(A)**. *SynCav1* significantly enhanced axonal length (um) and volume (um^3^) after 1 week **(B)** and increased both dendritic and axonal length (um) and volume (um^3^) at 3 weeks **(C)** as measured by Autoneuron, a tracing algorithm that measures 3D image volume stacks (Head et al., 2011). NPCs were stained for the MAP2 (dendrites and magenta), SMI 31 (axons and green), and Cav-1 (red). 40× magnification, Nikon confocal microscope. Data are expressed as mean ± S.E (*n* = 3, ^∗^*p* < 0.05, *t*-test).

As shown in Figure 1A, NPCs exhibited increased neuronal processes (i.e., increased axonal and dendritic growth as indicated by SMI 31 and MAP2 respectively, Figures 1B,C), while concurrently, the expression of Sox1, a stem cell marker, gradually decreased during this early stage of differentiation and were undetectable by day 7 days *in vitro* (DIV7) (Figures 1D,E). We have previously shown that *SynCav1* increases dendritic growth and arborization in primary rodent neurons *in vitro* (Head et al., 2011), therefore we tested whether these effects on neuroplasticity from *SynCav1* could be recapitulated in human NPCs. NPCs treated with *SynCav1* on DIV3 exhibited significant axonal growth (Figure 2A) (quantitation of length and volume, *n* = 3, *p* < 0.05, Figure 2B), with no effect dendritic growth after 1 week (DIV7). However, by week 3 (DIV21, 17 days post *SynCav1* treatment, Figure 2C) NPCs exhibited a significant increase in both MAP2-positive dendrites and SMI 31-positive axons (*n* = 3, *p* < 0.05, Figure 2D), akin to *SynCav1-*treated primary rodent neurons *in vitro* (Head et al., 2011). These findings demonstrate for the first time that *SynCav1* increases both dendritic and axonal growth in human neurons derived from iPSCs.

### *SynRac1CA* and *SynCdc42CA* Enhance Expression of P-Cav-1 and Axonal Growth

**FIGURE 3:**
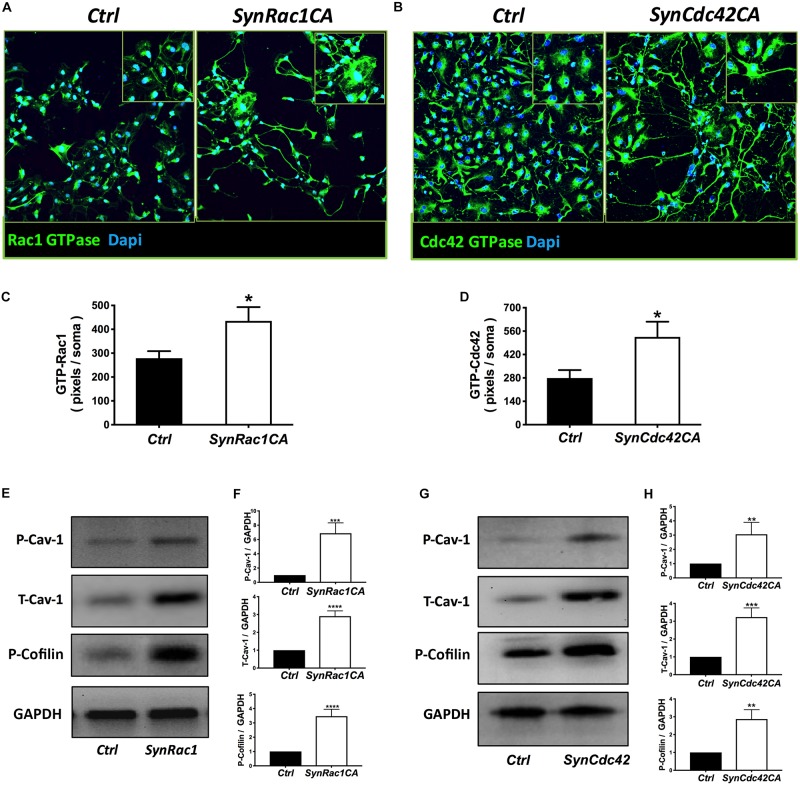
Constitutively active (CA) Rac1 and Cdc42 enhance expression of P-Cav-1, T-Cav-1 and P-Cofilin. NPCs were treated with *SynRac1CA* or *SynCdc42CA* (2 × 10^9^ IU/μl) and assayed for Rac1 GTPase (GTP-Rac1) **(A)** and Cdc42 GTPase (GTP-Cdc42) **(B)**, respectively. Quantitation of Rac1 GTPase **(C)** and Cdc42 GTPase **(D)**. Data are presented as total pixels (green) per soma (Dapi, blue). GTP-Rac1 **(E)** and GTP-Cdc42 **(G)** treated cell lysate were probed for P-Cav-1, T-Cav-1, and P-Cofilin (**F** for GTP-Rac; **H** for GTP-Cdc42). Data are expressed as mean ± S.E (*n* = 3, ^∗^*p* < 0.05, ^∗∗^*p* < 0.01, ^∗∗∗^*p* < 0.001, ^∗⁣∗⁣∗∗^*p* < 0.0001, One-way ANOVA).

**FIGURE 4:**
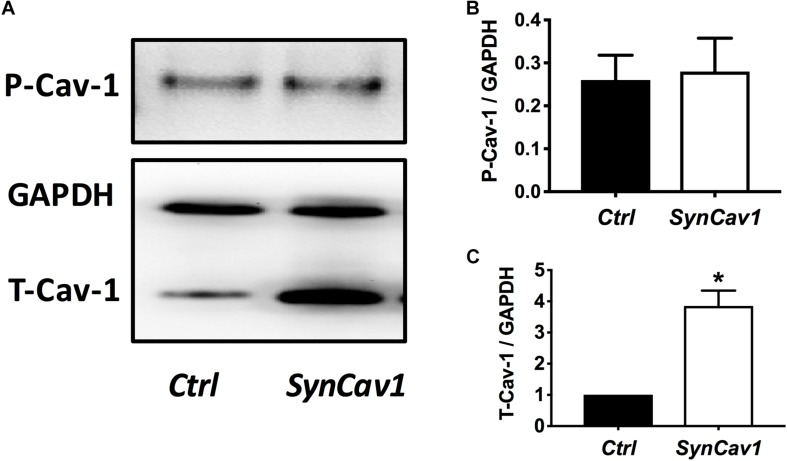
*SynCav1* increases T-Cav-1 but not P-Cav-1 in NPCs. **(A)** NPCs were treated with *SynCav1* (2 × 10^9^ IU/μl) for 72 hours followed by Western blot for P-Cav-1, T-Cav-1, and GAPH. Quantitation for P-Cav-1 and T-Cav-1 are shown in **(B**,**C)**, respectively. Data are expressed as mean S.E (*n* = 3, ^∗^*p* < 0.05, *t*-test).

**FIGURE 5:**
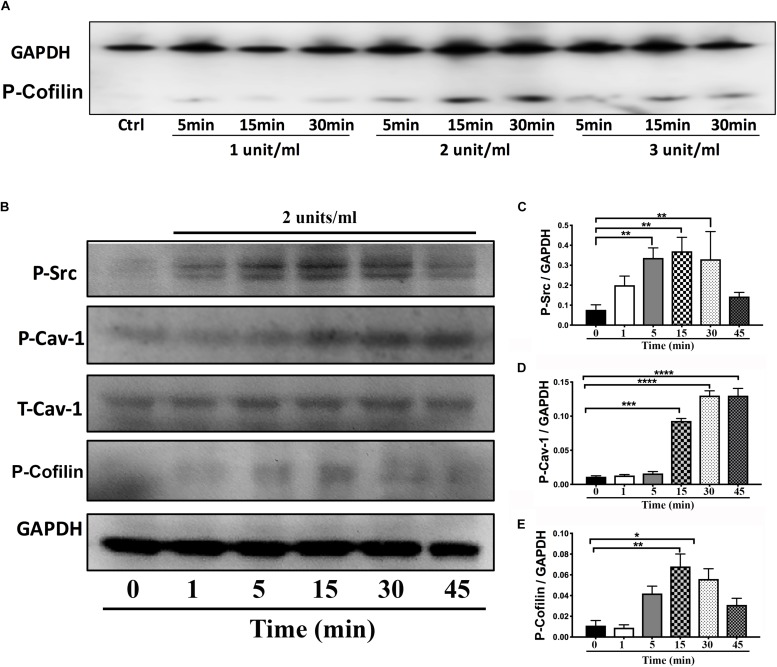
Time-course of P-Cav1 expression with the Rac1/Cdc42 activator in NPCs. **(A)** NPCs were incubated with three doses of the Rac1/Cdc42 activator (1–3 units/ml) and assayed for P-cofilin at three time points (5, 15, and 30 min) using Western blot. **(B)** NPCs were incubated with 2 units/ml of the activator followed by Western blot for P-Cav-1, T-Cav-1, P-Src, and P-cofilin at 1, 5, 15, 30, and 45 min. Quantitation is shown for P-Cav-1 **(C)**, P-Src **(D)**, and P-Cofilin **(E)**. Data are expressed as mean ± S.E (*n* = 3, ^∗^*p* < 0.05, ^∗∗^*p* < 0.01, ^∗∗∗^*p* < 0.001, ^∗⁣∗⁣∗∗^*p* < 0.0001, One-way ANOVA).

On DIV4, human NPCs were treated with constitutively active (CA) *SynRac1CA* or *SynCdc42CA* for an additional 3 days (DIV6). To confirm constitutively activation of Rac1 and Cdc42, antibodies to GTP-bound Rac1 or Cdc42 were used for immunofluorescence (IF). As shown in Figures 3A,B, a significant increase in GTP-bound Rac1 and Cdc42 was observed following *SynRac1CA* and *SynCdc42CA*, respectively (*n* = 3, *p* < 0.05, Figures 3C,D). Western blots analysis showed that Rac1CA and Cdc42CA resulted in downstream phosphorylation (i.e., inactivation) of cofilin [P-cofilin1(E5)], a protein that directly regulates actin dynamics (Figures 3E,G). Interestingly, Rac1CA and Cdc42CA also increased expression of total (T-) Cav-1 and phosphorylated (P-)Cav-1(Y14) (*n* = 3, *p* < 0.05, Figures 3F,H); surprisingly there was no increase in P-Cav-1 following *SynCav1* treatment (*n* = 3, *p* < 0.05, Figures 4A,B). We next stimulated NPCs with the Rac1/Cdc42 small molecule activator at 3 different doses. Maximal P-cofilin1 was measured at 2 unit/ml (Figure 5A). The small molecule activator also increased P-Cav-1 at 15, 30, and 45 min (Figure 5B), increased P-Src (Y416) at 1, 5, and 15 min (decreasing back to basal by 45 min), and increased P-cofilin1 at 5, 15, and 30 min. The increase in P-Cav-1 with *SynRac1CA* and *SynCdc42CA*, but not with *SynCav1*, suggests that detectable changes in P-Cav-1 occur early and transiently or only during an activated signaling pathway (i.e., activated Rac1, Cdc42).

### *SynCav1(Y14A)*-Treated NPCs Exhibit Stunted Axonal Growth During Early Stage of Differentiation

**FIGURE 6:**
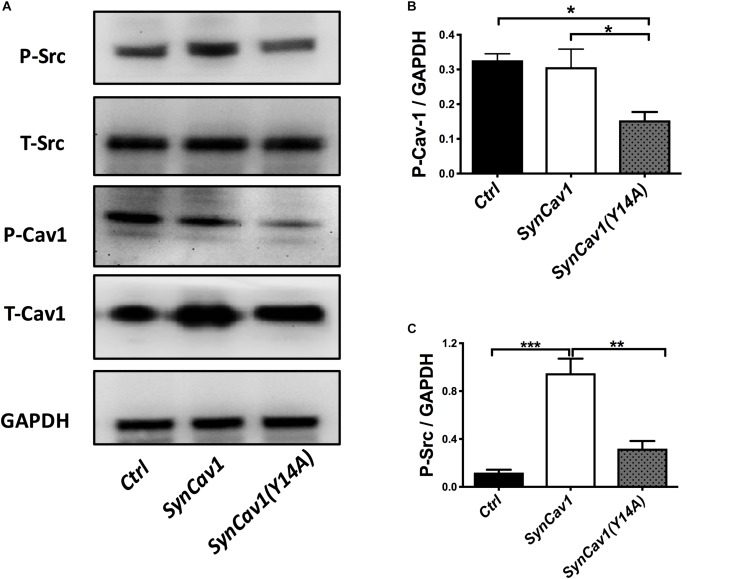
*SynCav1 (Y14A)* inhibits P-Cav-1 expression in NPCs. NPCs were incubated with *SynRFP, SynCav1*, or *SynCav1(Y14A)* (2 × 10^9^ IU/μl) on day 3. On week 2, NPCs were lysed and probed for P-Src(Y416), T-Src, P-Cav-1(Y14), T-Cav-1, and GAPDH. Quantitation for P-Src and P-Cav-1 are shown in **(B**,**C)**, respectively. Data are expressed as mean ± S.E (*n* = 3, ^∗^*p* < 0.05, ^∗∗^*p* < 0.01, ^∗∗∗^*p* < 0.001, One-way ANOVA).

**FIGURE 7:**
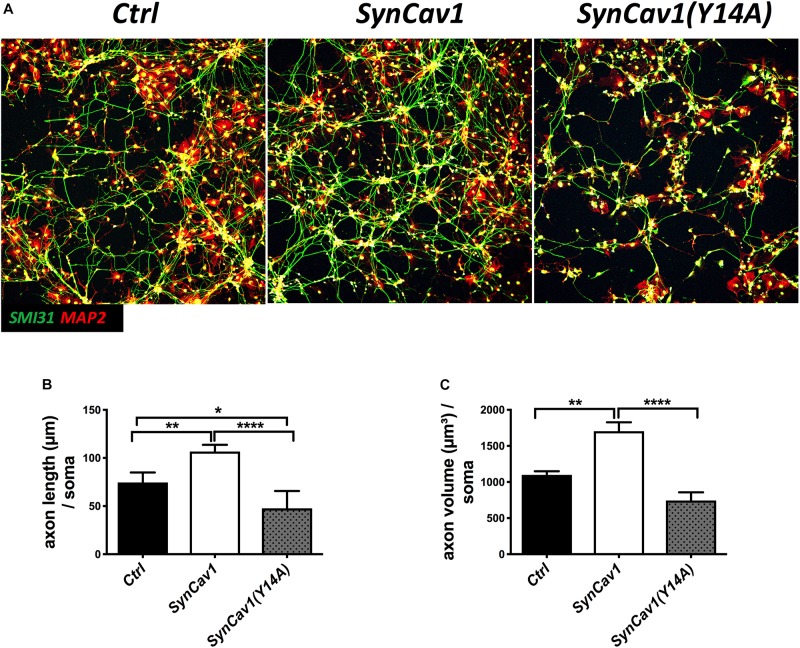
*SynCav1 (Y14A)* blunts axonal growth in NPCs during differentiation. **(A)** NPCs were treated with *Ctrl, SynCav1*, or *SynCav1(Y14A)* (2 × 10^9^ IU/μl) on day 3. On week 2, NPCs were stained for the dendritic marker MAP2 (red), SMI31 (green), and DAPI (blue). Quantitation of axonal length and volume are shown in **(B**,**C)**, respectively. Data are expressed as mean ± S.E (*n* = 3, ^∗^*p* < 0.05, ^∗∗^*p* < 0.01, ^∗∗∗∗^*p* < 0.0001, One-way ANOVA).

Neuronal progenitor cells were treated with a mutated Cav-1 construct [*SynCav1(Y14A)*] which prevents phosphorylation at Y14, resulting in a dominant negative Cav-1. At DIV3, NPCs were treated with *SynCav1* and *SynCav1(Y14A)* for 4 days. Western blot assay showed that both *SynCav1(Y14A)* and *SynCav1* significantly increased T-Cav-1, while *SynCav1(Y14A)* inhibited P-Cav-1, and P-Src expression (*n* = 3, *p* < 0.05, Figure 6). As shown in Figure 7A, NPCs showed a significant increase in axonal growth in *SynCav1*-treated cells compared to control, while *SynCav1(Y14A)* treated NPCs exhibited blunted axonal growth (i.e., stunted axonal length) (*n* = 3, *p* < 0.05, Figures 7B,C). These results demonstrated that *SynCav1*-promoted axonal growth is in part dependent upon Cav-1 phosphorylation of tyrosine (Y)14.

### Inhibition of P-Cav-1(Y14) Blunts Rac1/Cdc42-Mediated Axonal Growth and Differentiation of Human NPCs

**FIGURE 8:**
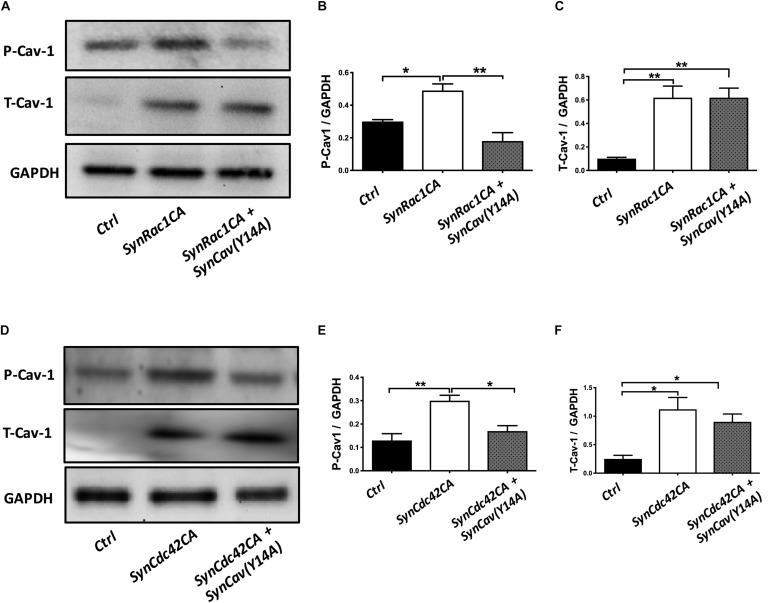
*SynCav1(Y14A)* prevents *SynRac1CA* and *SynCdc42CA*-mediated increased P-Cav-1 expression in NPCs. NPCs were treated with vectors (2 × 10^9^ IU/μl) as indicated on day 3 and WB was performed on week 2 to assay for P-Cav-1, T-Cav-1, and GAPDH. **(A)** Representative WB images of *Ctrl*, *SynRac1CA*, or *SynRac1CA + SynCav1(Y14A)* treated NPCs; Quantitation of P-Cav-1 and T-Cav-1 expression are shown in **(B**,**C)**, respectively. **(D)** Representative WB images of *Ctrl*, *SynCdc42CA*, or *SynCdc42CA + SynCav1(Y14A)* treated NPCs; Quantitation of P-Cav-1 and T-Cav-1 expression are shown in **(E**,**F)**, respectively. Data are expressed as the mean ± SE (*n* = 3, ^∗^*p* < 0.05, ^∗∗^*p* < 0.01, One-way ANOVA).

**FIGURE 9:**
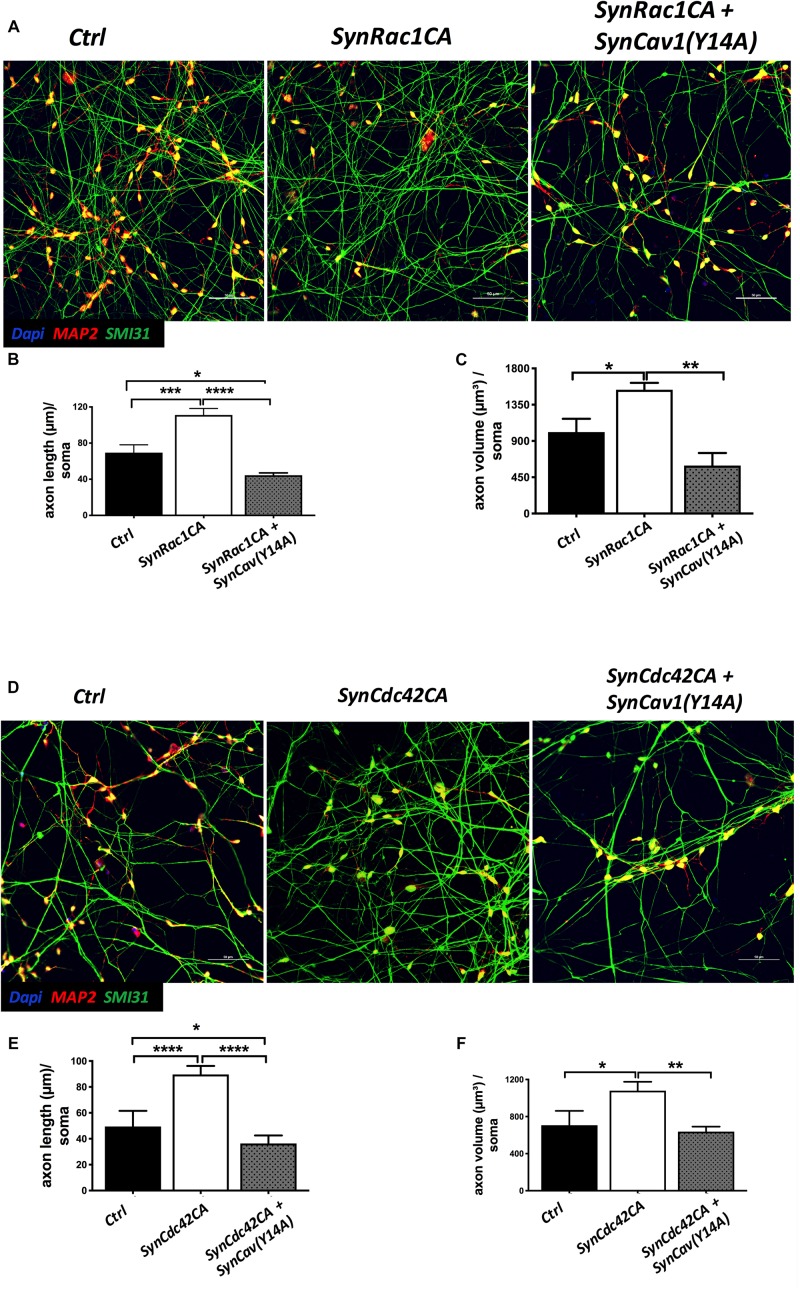
*SynCav1(Y14A)* suppresses *SynRac1CA* and *SynCdc42CA*-mediated axonal growth during NPC differentiation. NPCs were treated with vectors (2 × 10^9^ IU/μl) as indicated on day 3 and IF was performed on week 2. **(A)** Representative images of *Ctrl*, *SynRac1CA*, or *SynRac1CA + SynCav1(Y14A)* treated NPCs. Quantitation of axonal length (um) and volume (um^3^) are shown in **(B,C)**, respectively. **(D)** Representative images of *Ctrl*, *SynCdc42CA*, or *SynCdc42CA* plus *SynCav1(Y14A)* treated NPCs. Quantitation of axonal length (um) and volume (um^3^) are shown in **(E**,**F)**, respectively. NPCs were stained for MAP2 (red), SMI 31 (green) and DAPI (blue). Data are expressed as the mean ± SE (*n* = 3, ^∗^*p* < 0.05, ^∗∗^*p* < 0.01, ^∗∗∗^*p* < 0.001, ^∗⁣∗⁣∗∗^*p* < 0.0001, One-way ANOVA).

On DIV3, NPCs were treated with *SynRac1CA* or *SynCdc42CA*. As shown in Figure 8, *SynCav1(Y14A)* inhibited both *SynRac1CA* and *SynCdc42CA*-mediated P-Cav-1 expression (*n* = 3, *p* < 0.05). IF revealed that both *SynRac1CA* and *SynCdc42CA-*treated NPCs exhibited increased axonal length and volume, while cells co-transfected with *SynCav1(Y14A)* exhibited blunted axonal growth compared to control cells (*n* = 3, *p* < 0.05, Figure 9). These results suggest that Rac1/Cdc42-mediated axonal growth is in part dependent upon P-Cav-1.

## Discussion

The present study used differentiated human NPCs derived from iPSCs to demonstrate that (1) neuron-targeted Cav-1 (i.e., *SynCav1*) augments axonal growth during early stages of neuronal differentiation, a finding that extends previous work that *SynCav1* promotes neuroplasticity *in vitro* and *in vivo* (Head et al., 2010, 2011; Mandyam et al., 2017), (2) Cav-1-mediated axonal growth is abolished by inhibition of Cav-1 phosphorylation (Y14), and (3) axonal growth induced by Rac1/Cdc42 activation is in part dependent upon Cav-1 phosphorylation (Y14). In conclusion, Cav-1 phosphorylation is necessary for human neuronal axonal growth during early stages of neuronal differentiation.

Rho GTPases (e.g., RhoA, Rac1, and Cdc42) are key regulators of cytoskeletal dynamics, growth cone motility, and axonal guidance (Hall and Lalli, 2010; Dent et al., 2011) and are shown to regulate these processes at the plasma membrane (Baltierrez-Hoyos et al., 2012; Lim et al., 2014). While RhoA activation results in axonal growth retraction, Rac1, and Cdc42 activation promotes axonal growth. Dysfunctional Rac1/Cdc42 has been associated with aberrant synaptic plasticity and intellectual disability (Jiang et al., 2010; Tejada-Simon, 2015; Chen et al., 2016; Pengelly et al., 2016). On a subcellular level, evidence from non-neuronal cells has shown that Rac1 and Cdc42 co-localize and interact with Cav-1 at the plasma membrane microdomains (Baltierrez-Hoyos et al., 2012; Lim et al., 2014). Polarization of active Rac1 was lost in fibroblasts lacking Cav-1 (Williamson et al., 2015), and Cdc42 knockdown prevented ApoA-I-mediated increased Cav-1 in astrocytes (Kheirollah et al., 2014), findings which indicate an interplay between Cav-1, Rac1 and Cdc42-mediated signaling, and associated cellular effects. The present study shows that, in addition to increasing T-Cav-1, Rac1/Cdc42 activation also increases P-Cav-1 in NPCs 3 days post-treatment. Surprisingly, we did not observe elevated P-Cav-1 in *SynCav1* treated cells [which still increased T-Cav-1 as early as 24 hours post treatment (Supplementary Figure S2)]. The possible reason may be that increased P-Cav-1 occurs early and transiently during activated signaling events such as measured with constitutively Rac1(CA) and Cdc42(CA). Although not observed in the present study, *SynCav1-*mediated changes in P-Cav-1 may be detected immediately after receptor agonism or other activated signaling events (e.g., integrin activation, osmotic stress, and shear stress). However, the blunting of *SynCav1*-mediated axonal growth in the presence of P-Cav-1(Y14A) indicates that *SynCav1*-mediated axon growth is dependent upon its phosphorylation at Y14. These results lead us to postulate that P-Cav-1 may occur early and transiently following *SynCav1* treatment, resulting in the profound neuroplastic changes observed 1 and 3 weeks later. The present study builds upon these previous findings and is the first to definitively show in human neurons that activating signaling events (e.g., Rac1/Cdc42) that increase axonal growth is dependent upon P-Cav-1.

Previous research showed that Cdc42 activation enhances P-Src, while silencing Cdc42 prevents P-Src (Shen et al., 2008). Other work also found that Rac1 activates Src signaling in non-neuron cells (Fang et al., 2016). Because P-Cav-1(Y14) is mediated by Src family kinases (SFKs) (Li et al., 1996), the significant enhancement of P-Cav-1 with constitutively active Rac1/Cdc42 or with the Rac1/Cdc42 small molecule activator observed in the present study may be caused by the persistent activation of SFKs (Grande-Garcia et al., 2007). Therefore, we hypothesized that activation of Rac1/Cdc42 is dependent upon SFK-mediated P-Cav-1(Y14). By using *SynCav*1*(Y14A)*, we found that loss of P-Cav-1(Y14) blunted P-Src (Y416) Rac1/Cdc42-mediated axonal growth, indicating that Rac1/Cdc42-mediated axonal growth in early stages of differentiation is in part dependent on P-Src and P-Cav-1.

Our previous findings showed that *SynCav1* increased dendritic arborization in hippocampal neurons *in vivo* (Mandyam et al., 2017), which is consistent with the present study that *SynCav1* promotes dendritic growth in human neurons *in vitro*. Moreover, we also detected increased axonal growth as early as 1 week post *SynCav1* transfection, the first evidence that Cav-1 also regulates axonal growth. These findings extend previous work by others that MLRs and MLR-associated proteins (such as Cav-1) provide subcellular and molecular signaling complexes essential for axonal growth and guidance (Guirland and Zheng, 2007; Yamashita and Kuruvilla, 2016; Batty et al., 2017). The present study attempted to better elucidate the molecular mechanism(s) underlying Cav-1-mediated neuronal growth. Previous research already demonstrated that even subtle changes in axon connectivity and neuronal network formation may cause various neurological disorders ranging from Down Syndrome to Autism. Because Cav-1 phosphorylation is essential for *SynCav1-*promoted axonal growth in early-stage of differentiation, disruption or imbalances in P-Cav-1 may have implications for neurological diseases or during aberrant neuronal development.

Endocytosis and intracellular trafficking are critical for neuritogenesis, axonal guidance and maturation (Winckler and Yap, 2011), and are dependent upon membrane lipid composition (Lauwers et al., 2016). Emerging evidence shows that MLRs and Cav-1 are necessary for neuritogenesis (Shikanai et al., 2018), axonal guidance, and synaptogenesis (i.e., formation of new synapses) (Head et al., 2014; Egawa et al., 2016, 2017). We have previously demonstrated that *SynCav1* delivery to the hippocampus increased total excitatory type I synapses, multiple synapse forming boutons, LTP, and axonal myelination, all of which are ultrastructural indicators of axonal function and synaptic plasticity (Egawa et al., 2017). Although we did not test whether P-Cav-1 affects axonal transport of vesicles and/or cellular cargoes, the present study does show that NPCs treated with *SynCav1(Y14A)* resulted in profound impaired axonal growth in contrast to *SynCav1*, which significantly augmented axonal growth. Recent work demonstrated that P-Cav-1 facilitates microRNA insertion into extracellular vesicles by regulating RNA binding proteins in non-neuronal cells (Lee et al., 2019). Because P-Cav-1(Y14) and Src activation are linked to actin remodeling, endocytosis (Shi and Sottile, 2008), vesicular transport (Coelho-Santos et al., 2016), and trafficking (Cao et al., 2002, 2004; Salanueva et al., 2007; Chen et al., 2014) in non-neuronal cells, we are currently testing the hypothesis that P-Cav-1(Y14) may facilitate early stage signaling events necessary for axonal transport of vesicles to and from the presynaptic active zone as well as secretion of pro-growth neuronal microvesicles, subcellular events which are necessary for axonal growth and guidance, and synaptic maintenance.

In summary, these results show that Cav-1 phosphorylation is critical for axonal growth. Furthermore, because increased Cav-1 phosphorylation occurred with constitutively active Rac1 and Cdc42, but not with *SynCav1* alone, suggests that this post-translational event may occur early and transiently (i.e., signal transduction) in response to extracellular pro-growth signaling cues. The present study also demonstrated that *SynCav1*-promoted axonal growth is dependent upon P-Cav-1(Y14). Moreover, inhibition of endogenous phosphorylation of Cav-1 even in the presence of increased T-Cav-1 (i.e., *SynCav1*) results in aberrant and blunted axonal growth. In conclusion, targeting Cav-1, specifically Cav-1 phosphorylation in neurons, may serve as a novel therapeutic target to promote axonal growth during early-stage differentiation in the setting of neurodevelopmental disorder such as Down Syndrome or serve to promote functional neuroplasticity after CNS injury (ischemic or traumatic) and in neurodegenerative diseases (AD, ALS, and multiple sclerosis).

## Data Availability

The datasets generated for this study are available on request to the corresponding author.

## Author Contributions

SW wrote the manuscript and carried out the cell culture, immunofluorescence microscopy, Western blot, and data analyses. ZZ carried out the cell culture and Western blot. AA-Q provided the NPCs and assisted in cell culture. JL assisted in cell culture and Western blot. CD generated and provided the Rac1 and Cdc42 constructs. DR, PP, and HP assisted in editing and in experimental design. BH conceived the study, assisted in data analyses, provided the phospho-mutant Cav1 construct, and wrote the manuscript.

## Conflict of Interest Statement

HP and BH are scientific founders of CavoGene LifeSciences LLC and hold equity interest in the company. The remaining authors declare that the research was conducted in the absence of any commercial or financial relationships that could be construed as a potential conflict of interest.

## Supplementary Material

FIGURE S1 Representative tracing images using Autoneuron, which measures 3D image volume stacks (MBF Bioscience) generated by Neurolucida as previously described (Head et al., 2011).

FIGURE S2 Time course of *SynCav1*-mediated increase in total Cav-1 protein expression at 24, 48, and 72 h. Upper band, GAPDH; lower band, Cav-1. SC represents *SynCav1* treatment.

## Abbreviations

AAV: adeno-associated virus
ANOVA: analysis of variance
Cdc42: cell division control protein 42 homolog
LTP: long-term potentiation
Lv: lentivirus
MAP2: microtubule associated protein 2
MLRs: membrane/lipid rafts
NMDAR: N-methyl -D-aspartate receptor
Rac1: Ras-related C3 botulinum toxin substrate 1
*SynCav1(Y14A)*: tyrosine to alanine point mutation at residue 14
*SynCav1*: synapsin caveolin-1
*SynCdc42CA*: synapsin constitutively active Cdc42
*SynGFP*: synapsin green fluorescent protein
*SynRac1CA*: synapsin constitutively active Rac1
SynRFP: synapsin red fluorescent protein
Trk: tropomyosin receptor kinase B.
