# Supplementary figures and images for: Caveolin-1 Phosphorylation Is Essential for Axonal Growth of Human Neurons Derived From iPSCs
Source: Front Cell Neurosci. 2019 Jul 17;13:324. doi: 10.3389/fncel.2019.00324 (PMC6650578; doi:10.3389/fncel.2019.00324)

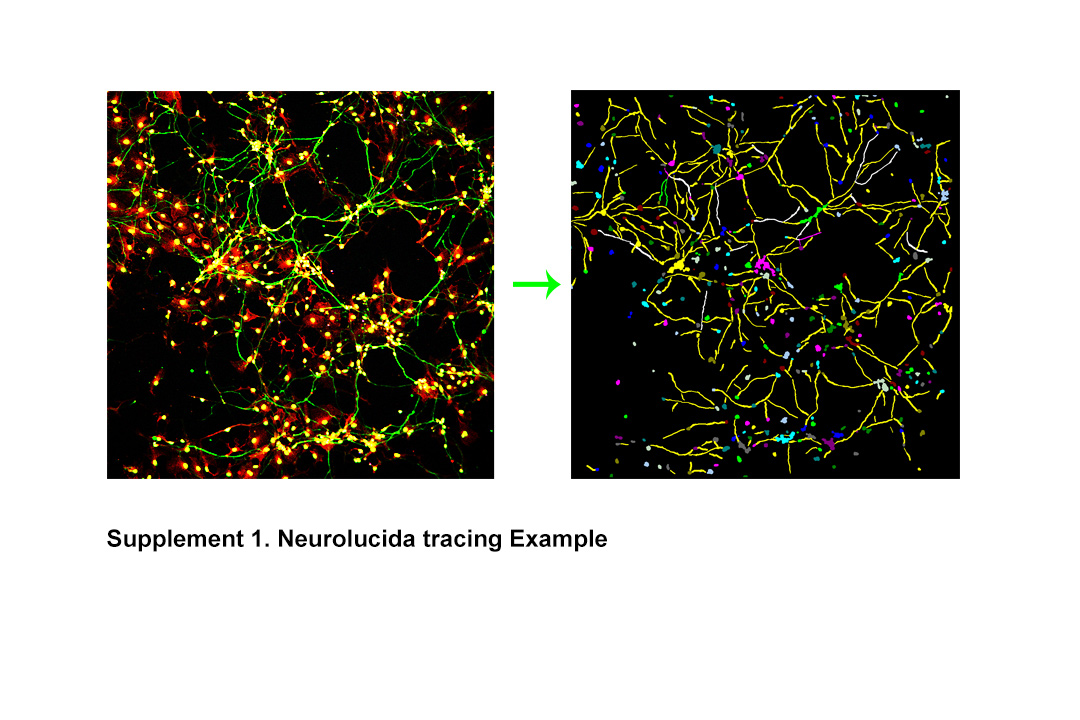

Supplement: FIGURE S1 — Representative tracing images using Autoneuron, which measures 3D image volume stacks (MBF Bioscience) generated by Neurolucida as previously described (Head et al., 2011). [file Image_1.JPEG]

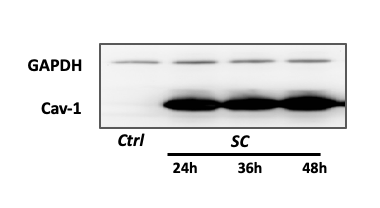

Supplement: FIGURE S2 — Time course of SynCav1-mediated increase in total Cav-1 protein expression at 24, 48, and 72 h. Upper band, GAPDH; lower band, Cav-1. SC represents SynCav1 treatment. [file Image_2.TIFF]
